# Impact of Sarcoidosis on In-hospital Outcomes Among Patients with Atrial Fibrillation: A Nationwide Readmissions Database Analysis

**DOI:** 10.19102/icrm.2024.15035

**Published:** 2024-03-15

**Authors:** Min Choon Tan, Qi Xuan Ang, Yong Hao Yeo, Boon Jian San, Ramzi Ibrahim, Sze Jia Ng, Jian Liang Tan, Jasjit Walia, Addi Suleiman, Joaquim Correia

**Affiliations:** 1 Department of Internal Medicine, New York Medical College at Saint Michael’s Medical Center, Newark, NJ, USA; 2 Department of Cardiovascular Medicine, Mayo Clinic, Phoenix, AZ, USA; 3 Department of Internal Medicine, Sparrow Health System and Michigan State University, East Lansing, MI, USA; 4 Department of Internal Medicine/Pediatrics, Beaumont Health, Royal Oak, MI, USA; 5 AIMST University, Bedong, Malaysia; 6 Department of Internal Medicine, University of Arizona—Banner University Medical Center, Tucson, AZ, USA; 7 Department of Internal Medicine, Crozer-Chester Medical Center, Upland, PA, USA; 8 Department of Cardiovascular Medicine, Hospital of the University of Pennsylvania, Philadelphia, PA, USA; 9 Department of Cardiovascular Medicine, New York Medical College at Saint Michael’s Medical Center, Newark, NJ, USA

**Keywords:** Atrial fibrillation, hospital outcomes, sarcoidosis

## Abstract

Sarcoidosis is a disease that involves multiple organs, including the cardiovascular system. While cardiac sarcoidosis has been increasingly recognized, the impact of sarcoidosis on atrial fibrillation (AF) is not well established. This study aimed to analyze the impact of sarcoidosis on in-hospital outcomes among patients who were admitted for a primary diagnosis of AF. Using the all-payer, nationally representative Nationwide Readmissions Database, our study included patients aged ≥18 years who were admitted for AF between 2017–2020. We stratified the cohort into two groups depending on the presence of sarcoidosis diagnosis. The in-hospital outcomes were assessed between the two groups via propensity score analysis. A total of 1031 (0.27%) AF patients with sarcoidosis and 387,380 (99.73%) AF patients without sarcoidosis were identified in our analysis. Our propensity score analysis of 1031 (50%) patients with AF and sarcoidosis and 1031 (50%) patients with AF but without sarcoidosis revealed comparable outcomes in early mortality (1.55% vs. 1.55%, *P* = 1.000), prolonged hospital stay (9.51% vs. 9.70%, *P* = .874), non-home discharge (7.95% vs. 9.89%, *P* = .108), and 30-day readmission (13.29% vs. 13.69%, *P* = .797) between the two groups. The cumulative cost of hospitalization was also similar in both groups ($12,632.25 vs. $12,532.63, *P* = .839). The in-hospital adverse event rates were comparable in both groups. Sarcoidosis is not a risk factor for poorer in-hospital outcomes following AF admission. These findings provide valuable insights into the effectiveness of the current guideline for AF management in patients with concomitant sarcoidosis and AF.

## Background

Sarcoidosis is a systemic inflammatory disorder that affects multiple organs.^1^ The deposition of granulomas in cardiac tissue may predispose an individual to atrial or ventricular arrhythmias, conduction system abnormalities, and heart failure.^2–5^ Atrial fibrillation (AF) remains the most common type of supraventricular arrhythmia in patients with sarcoidosis, with a prevalence of 12%–18%.^6,7^ However, data on the impact of sarcoidosis on in-hospital outcomes among those with AF are not well established.

## Methods

We queried the all-payer, nationally representative Nationwide Readmissions Database to analyze patients aged ≥18 years who were admitted for AF between January and November during each calendar year from 2017–2020. We stratified the cohort into two groups based on the presence or absence of sarcoidosis diagnosis using the International Classification of Diseases, Tenth Revision, Clinical Modification (ICD-10-CM) diagnosis code D86. The main outcomes examined were: (1) in-hospital adverse events, (2) length of stay, (3) discharge disposition, (4) 30-day readmission rate, (5) early mortality (mortality during index hospitalization and readmission), and (6) cumulative cost of hospitalization. As the Nationwide Readmissions Database provides de-identified patient data and is publicly accessible, institutional review board approval was not required for this study.

Continuous data were summarized as mean with standard deviation values or median with interquartile range (Q1, Q3) values depending on their distribution; differences between groups were tested using Wilcoxon rank-sum tests. Categorical data were summarized as counts and percentages; differences between groups were tested using Pearson’s chi-squared test. All tests were two-sided with *P* ≤ .05, indicating statistical significance. Statistical analyses were conducted using Stata version 12.1 (Stata Corporation, College Station, TX, USA). To identify the association between sarcoidosis and in-hospital outcomes, weighted propensity score matching was first performed with a caliper of 0.2 with a nearest-neighbor ratio of 1:1 for each hospital outcome. Then, all variables outlined in **Table 1**, including sarcoidosis, were included in the univariable analysis to study their association with the outcome variables listed in **Table 2**. Those relevant variables with *P* < .1 were included in a multivariable model for conditional logistic regression analyses.

## Results

We identified a total of 388,411 patients hospitalized with AF in the United States from 2017–2020. Our respective cohort consisted of 1031 (0.27%) AF patients with sarcoidosis and 387,380 (99.73%) AF patients without sarcoidosis. **Table 1** depicts the baseline characteristics of our patient cohort. AF patients with sarcoidosis were younger and had greater prevalence rates of chronic kidney disease, chronic pulmonary disease, heart failure, non-ischemic cardiomyopathy, obstructive sleep apnea, peripheral vascular disease, prior implantable cardioverter-defibrillator placement, and pulmonary hypertension. A propensity score analysis was performed, which yielded 1031 (50%) patients with AF and sarcoidosis and 1031 (50%) patients with AF but without sarcoidosis. There was no difference in early mortality (1.55% vs. 1.37%, *P* = 1.00), prolonged hospital stay ≥7 days (9.51% vs. 9.03%, *P* = .87), non-home discharge (7.95% vs. 11.18%, *P* = .11), or 30-day readmission (13.29% vs. 12.86%, *P* = .80) when compared among patients with and without sarcoidosis who were admitted for AF **(Table 2)**. The cumulative cost of hospitalization was also similar in both groups ($12,632.25 vs. $12,532.63, *P* = .84). There was no significant difference in rates of in-hospital adverse events between both groups, including acute heart failure (16.68% vs. 17.05%, *P* = .46), cardiogenic shock (0.48% vs. 0.47%, *P* = .57), cardiac arrest (0.48% vs. 0.23%, *P* = 1.00), cerebral infarct (0.48% vs. 0.30%, *P* = .71), pulmonary edema (1.36% vs. 0.79%, *P* = .08), acute kidney injury (12.71% vs. 11.63%, *P* = .84), and venous thromboembolism (0.97% vs. 0.83%, *P* = .64). Further subgroup analysis revealed that sarcoidosis was not independently associated with greater odds for any in-hospital adverse events among patients with AF **(Table 3)**. Additional subgroup analysis demonstrated decreases of 1.21% and 7.57% in the yearly AF-related admission among the sarcoidosis and non-sarcoidosis cohorts, respectively.

## Discussion

This study is the first to provide insights on in-hospital adverse events and 30-day readmission rates among patients with and without sarcoidosis who were admitted for AF in a real-world setting. Despite an increased risk of AF and greater comorbidity burden among patients with sarcoidosis, our study suggests that patients with sarcoidosis and AF did not experience poorer in-hospital outcomes when compared to patients without sarcoidosis.^6^ AF in sarcoidosis was hypothesized to be caused by atrium granuloma leading to scarring and by sarcoid involvement of the lungs and left ventricle, resulting in increased end-diastolic pressure.^6,8^ The non-inferior outcomes observed in sarcoidosis provide a reflection of contemporary real-world data on the effectiveness of AF management in sarcoidosis by early diagnosis and treatment of cardiac sarcoidosis as well as early intervention, including rate control, rhythm control, or even catheter ablation, as per guideline in all AF patients regardless of the underlying etiology.^5,9,10^ Our study also demonstrates that sarcoidosis is not an independent risk factor of in-hospital adverse events during hospitalization.

### Limitations

It is important to acknowledge a few main limitations of this study. First, as with most large administrative database studies, the main limitation includes potential miscoding in primary diagnoses and under-reporting of secondary diagnoses. Next, the out-of-hospital deaths that occurred prior to readmission were not recorded, which limits our early mortality to in-hospital mortality. Furthermore, clinical information, including the duration of AF, cardiac involvement of sarcoidosis, and anti-arrhythmic medications, was not available in the database, limiting our attempts to explore the impact of these clinical variables on hospital outcomes.

## Conclusion

Our study suggests that sarcoidosis is not associated with poorer hospital outcomes among patients hospitalized for AF. These findings provide valuable insights into the effectiveness of the current guideline for AF management in patients with concomitant sarcoidosis and AF.

## Figures and Tables

**Table 1:** Baseline Characteristics of the Patient Cohort

	AF Patients with Sarcoidosis		AF Patients Without Sarcoidosis		*P* Value
	n	%	n	%	
No. of admissions	1031	0.3	387,380	99.7	
Baseline characteristics					
Age, mean (SD), years	67.70 (11.43)		71.63 (12.45)		<.01
Female sex	630	61.1	200,145	51.7	<.01
Anemia	45	4.4	16,178	4.2	.76
Catheter ablation for AF	50	4.9	14,429	3.7	.06
CHA_2_DS_2_-VASc score, points	3 (2)		3 (2)		.14
Chronic kidney diseases	266	25.8	76,206	19.7	<.01
Chronic liver disease	45	4.4	12,604	3.3	.05
Chronic pulmonary disease	376	36.5	94,541	24.4	<.01
Coagulation disorder	58	5.6	18,700	4.8	.23
Coronary artery disease	293	28.4	127,991	33.0	<.01
Heart failure	205	19.9	55,560	14.3	<.01
Hyperlipidemia	547	53.1	213,010	55.0	.21
Hypertension	841	81.6	314,372	81.2	.73
Malignancy	52	5.0	19,769	5.1	.93
Non-ischemic cardiomyopathy	320	31.0	86,911	22.4	<.01
Obstructive sleep apnea	266	25.8	70,398	18.2	<.01
Peripheral vascular disease	131	12.7	39,930	10.3	.01
Prior myocardial infarction	77	7.5	34,050	8.8	.13
Prior coronary artery bypass graft	40	3.9	26,778	6.9	<.01
Prior ICD	68	6.6	11,625	3.0	<.01
Prior PPM	65	6.3	26,288	6.8	.54
Prior percutaneous coronary intervention	88	8.5	41,436	10.7	.03
Prior stroke/transient ischemic attack	100	9.7	49,395	12.8	<.01
Pulmonary hypertension	172	16.7	33,450	8.6	<.01
Valvular heart disease	203	19.7	82,869	21.4	.18
Charlson Comorbidity Index (%), points					<.01
0	149	14.4	90,095	23.3	
1	236	22.9	98,240	25.3	
≥2	646	62.7	199,045	51.4	
Elixhauser Comorbidity Score, points					<.01
<4	248	24.1	129,181	33.3	
≥4	783	75.9	258,199	66.7	

*Abbreviations:* AF, atrial fibrillation; ICD, implantable cardioverter-defibrillator; PPM, permanent pacemaker; SD, standard deviation.

**Table 2:** In-hospital Outcomes of AF Patients with Sarcoidosis Versus Without Sarcoidosis via Propensity Score Analysis

	AF Patients with Sarcoidosis (n = 1031)	AF Patients Without Sarcoidosis (n = 1031)	*P* Value
	%	%	
Hospital outcomes			
Early mortality	1.55	1.55	1.000
Prolonged hospital stay	9.51	9.70	.874
Non-home discharge	7.95	9.89	.108
30-day readmission	13.29	13.69	.797
Cost of hospitalization	$12 632.25	$12 532.63	.839
In-hospital adverse events			
Acute heart failure	19.50	20.63	.456
Cardiogenic shock	0.48	0.68	.566
Cardiac arrest	0.48	0.48	1.000
Cerebral infarction	0.57	0.45	.706
Pulmonary edema	1.36	0.58	.082
Acute kidney injury	12.71	12.42	.836
Venous thromboembolism	0.97	0.78	.638

*Abbreviation:* AF, atrial fibrillation.

**Table 3:** Analysis of Hospital Outcomes of AF Patients with Versus Without Sarcoidosis

	OR	Lower CI	Upper CI	*P* Value
Early mortality	1.00	0.48	2.10	1.000
Prolonged hospital stay (≥7 days)	0.98	0.71	1.33	.874
Non-home discharge	0.77	0.56	1.06	.108
30-day readmission	0.97	0.75	1.24	.797
Acute heart failure	0.89	0.67	1.20	.456
Cardiogenic shock	0.71	0.23	2.25	.566
Cardiac arrest	1.00	0.29	3.45	1.000
Ischemic stroke	1.33	0.30	5.96	.706
Pulmonary edema	2.33	0.90	6.07	.082
Acute kidney injury	1.03	0.78	1.35	.836
Venous thromboembolism	1.25	0.49	3.17	.638

*Abbreviations:* AF, atrial fibrillation; CI, confidence interval; OR, odds ratio.
